# Prevalence of self-medication among university students diagnosed with temporomandibular disorders

**DOI:** 10.22514/jofph.2024.027

**Published:** 2024-09-12

**Authors:** Stefany Joaquina Sousa Farias, Alexia Guimarães Ramos, Evelyn Mikaela Kogawa, Erica Negrini Lia, Rodrigo Antonio de Medeiros

**Affiliations:** ^1^Department of Dentistry, School of Health Sciences, University of Brasília (UnB), 70910-900 Brasília, DF, Brazil

**Keywords:** Self-medication, Temporomandibular disorders, Temporomandibular joint dysfunction syndrome, Oral health

## Abstract

Temporomandibular disorders (TMD) usually affect the stomatognathic system and can be symptomatic. Patients often self-medicate to relieve symptoms, and this can increase the risk of complications such as adverse drug reactions, overdose, physical and psychological dependence, and delay of appropriate treatment. It is important for dentists to know the prevalence of self-medication to investigate this condition in their patients, thus the primary aim of this study was to estimate the prevalence of self-medication among university students with no TMD, non-painful TMD and painful TMD and the secondary aim was to assess association with independent factors. An online questionnaire was used to assess TMD symptoms (DC/TMD—Diagnostic Criteria for Temporomandibular Disorders: Clinical Protocol and Assessment Instruments) and self-medication practices (QAM/TMD—Questionnaire on the practice of self-medication associated with mandibular disorders). Qualitative data were analyzed using the Fisher’s exact test and chi-square test, while the relationships between qualitative and quantitative data were examined using Spearman’s rho correlation test. The level of statistical significance was set at *p*-value < 0.05. In total, 179 university students completed the questionnaire, of which 113 (63.1%) reported TMD symptoms. The majority (84.9%) practiced mild self-medication, and only 12.3% of patients with TMD symptoms practiced moderate or severe self-medication. Students with painful TMD are more likely to self-medicate than those that remain unaffected or exhibit non-painful TMD.

## 1. Introduction

Temporomandibular disorders (TMD) affect the temporomandibular joint, masticatory muscles, and associated structures [1, 2], and have a multifactorial etiology that includes environmental, biological, emotional, social and cognitive factors [3]. Previous research has demonstrated that the COVID-19 pandemic has had a substantial impact on the mental health and quality of life of university students. As a result, there has been an increase in the prevalence of TMD in this population [4, 5]. Patients with TMD may remain asymptomatic or exhibit various signs and symptoms such as limitation of mouth opening and contralateral movements; crackling or clicking sounds during mandibular movement; functional limitation; and orofacial pain [2]. Not all TMD patients require treatment, with spontaneous resolution of symptoms occurring in 40%. Initial treatment approaches for those who experience pain and functional limitations should aim to control these symptoms [3]. However, some forms of TMD can also require complementary pharmacological treatment with analgesics (non-opioids and opioids); non-steroidal anti-inflammatory drugs (NSAIDs); benzodiazepines; muscle relaxants; or tricyclic antidepressants [6, 7]. Effective pain management may require multiple treatment methods, highlighting the significance of follow-up and identifying appropriate therapeutic protocols by qualified professionals [6].

However, self-medication is a common practice among TMD patients seeking relief from symptoms such as pain. In a preliminary study conducted in São Paulo, almost 50% of affected individuals reported using this method [8]. Self-medication is the occasional or continuous selection and use of medications without the guidance of a qualified health professional [9, 10]. It is associated with several risks, including incorrect use, non-compliance with the correct dosage, imprecise treatment duration, physical and psychological drug dependence, use of medications in the presence of contraindications, and failure to address the root cause of the problem, thus delaying appropriate treatment [10].

In the literature, however, there is limited information on the associations between the practice of self-medication and TMD symptoms. Thus, this study aims to estimate the prevalence of self-medication among university students with no TMD, non-painful TMD and painful TMD. Additionally, the study aims to assess the association with independent factors. The hypothesis was that there would be no association between the practice of self-medication and the type of TMD symptoms.

## 2. Materials and methods

### 2.1 Study design

This voluntary cross-sectional study using primary data. There was no interference from researchers in the selection of participants.

### 2.2 Setting

The study was conducted at the School of Health Sciences (FS), University of Brasília (UnB), between May and June 2022.

### 2.3 Participants

The study sample consisted of students over 18 years of age who were currently enrolled in Nursing, Pharmacy, Nutrition, Dentistry or Public Health courses at FS, UnB. The exclusion criteria were pre-existing chronic facial and non-facial pain. In addition, students who completed the questionnaire incompletely or incorrectly, which could have led to inaccurate classification of findings and identification, were excluded from the study to minimize the risk of bias. The study population was invited to participate in the study via e-mail and social media.

### 2.4 Data collection

Participants were given a web link to a questionnaire allowing data collection on the patient’s sociodemographic characteristics and general health (a question about a previously diagnosed chronic condition). Additionally, it also included the DC/TMD (Diagnostic Criteria Questionnaire for Temporomandibular Disorders: Clinical Protocol and Assessment Instruments) [11] and QAM/TMD (questionnaire on the practice of self-medication associated with mandibular disorders) [12] questionnaires.

The DC/TMD evaluates the presence of TMD symptoms using objective questions composed of different questionnaires [11]. The version in Brazilian Portuguese published by INfORM (International Network for Orofacial Pain and Related Disorders Methodology) was utilized. The participants answered the Symptom Questionnaire. Due to local health restrictions at the time, no further clinical examinations were performed.

A specialist analyzed the completed questionnaires and the participants were categorized into three groups based on symptoms experienced in the last 30 days: (a) no TMD; (b) painful TMD (determined using the first two questions of related to the presence of pain and headaches); and (c) non-painful TMD (determined using questions related to the presence of joint sounds, closed locking and/or locking) [4].

The QAM/DTM was used to assess the practice of self-medication for TMD [12]. The questionnaire had 34 questions with scores between 1 and 5, with 5 representing the most favorable response to self-medication. Participants were divided into 3 groups (*i.e.*, mild: 34–81 points; moderate: 82–103 points; or severe: 104–170 points) based on exposure to self-medication. The specific drug classes used were recorded using the statement numbers 10 (“When I experience facial pain, I take any medicine that can reduce it as I do not know the cause”); 11 (“I take analgesics on my own when I experience facial pain”); 12 (“I take muscle relaxants on my own when I experience facial pain”), and 13 (“I take anti-inflammatory drugs on my own when I experience facial pain”).

### 2.5 Study size

The sample calculation, performed using OpenEpi 2.3, considered the frequency of self-medication in Brazilian students to be 89% [13]. The confidence interval was set at 90%, with 5% and 1.0 confidence limits for design purposes. The estimated sample size was 101 students with TMD symptoms in a population of 1967 active students at FS, UnB.

### 2.6 Statistical analysis

All collected data were compiled in Microsoft Office Excel spreadsheets and statistical analysis was performed using the IBM SPSS (Statistical Package for Social Sciences, v26; IBM Corporation, Armonk, NY, USA) software. A chi-square test or Fisher’s exact test was used to investigate possible associations between gender, TMD symptoms, and self-medication, while the Spearman’s rho correlation test was used to examine the relationship between TMD symptoms and questions 10, 11, 12 and 13 (as detailed above). The level of statistical significance was set at 5%.

## 3. Results

The questionnaire had 200 respondents, of which 21 were excluded based on the criteria mentioned above (identification of pre-existing chronic facial and/or non-facial pain: 14 participants; students that incompletely or incorrectly completed the questionnaire: 7 participants). The final sample consisted of 179 participants, as shown in detail in Table 1. The participants had a mean age of 22.3 ± 3.1 years.

**Table 1.** Demographics (N = 179).

Variables		N	%
Gender			
	Female	138	77.1
	Male	41	22.9
Undergraduate course			
	Dentistry	88	49.1
	Nursing	33	18.4
	Nutrition	24	13.4
	Pharmacy	21	11.7
	Collective Health	13	7.2

Approximately 113 students exhibited TMD symptoms, of which 87 experienced pain and 26 reported no pain. All 179 students who completed the questionnaire reported practicing self-medication, and the QAM/DTM classification showed that 152 self-medicated mildly, 26 self-medicated moderately, and 1 self-medicated severely. Analgesics were the most used drugs, with 20.7% of the study sample and 31% of patients with symptomatic TMD reporting usage.

The chi-square test showed an association between gender and TMD symptoms (χ^2^ (2) = 8393; *p*-value = 0.015), with pain being significantly associated with the female gender (*p*-value < 0.001; Table 2). The Fisher’s exact test showed no association between sex and self-medication (χ^2^ (2) = 2567; *p*-value = 0.338; Table 2) and a significant association between self-medication and TMD symptoms (χ^2^ (4) = 11,055; *p*-value = 0.01). A higher prevalence of moderate self-medication was observed among those with painful TMD symptoms (*p*-value < 0.001; Table 3).

**Table 2.** Frequency (percentage) table showing the association between gender and various analysis variables (Chi-square and Fisher’s exact test, *p*-value < 0.05).

Variables		Gender		*p*-value
		Female	Male	
TMD Symptoms^†^				
	No TMD	44 (66.7%)	22 (33.3%)	0.015*
	Non-painful TMD	19 (73.1%)	7 (26.9%)	
	Painful TMD	75 (86.2%)	12 (13.8%)	
Self-medication^‡^				
	Mild	114 (75.0%)	38 (25.0%)	0.338
	Moderate	23 (88.5%)	3 (11.5%)	
	Severe	1 (100.0%)	0 (0.0%)	

*p < 0.05, showing statistically significant difference; ^†^Chi-square Test; ^‡^Fisher’s exact test. TMD: Temporomandibular disorders.

**Table 3.** Frequency (percentage) table showing the association between TMD symptoms and self-medication (Fisher’s exact test, *p*-value < 0.05).

Self-medication	TMD Symptoms			*p*-value
	No TMD	Non-painful TMD	Painful TMD	
Mild	61 (40.1%)	25 (16.4%)	66 (43.5%)	0.010*
Moderate	5 (19.2%)	1 (3.8%)	20 (77.0%)	
Severe	0 (0.0%)	0 (0.0%)	1 (100.0%)	

*p < 0.05. TMD: Temporomandibular disorders.

The Spearman’s rho correlation test showed a positive association between TMD symptoms and the use of any medication, analgesics, muscle relaxants, and anti-inflammatory agents in the presence of facial pain (Table 4).

**Table 4.** Spearman’s rho correlation test showing association between different classes of medication and the presence of facial pain and TMD symptoms (*p*-value < 0.05).

Variables	Any medication	Analgesics	Muscle relaxants	Anti-inflammatory agents
TMD Symptoms	*r *= 0.250 *p* < 0.001*	*r *= 0.278 *p *< 0.001*	*r* = 0.301 *p *< 0.001*	*r* = 0.253 *p *< 0.001*

r = Correlation coefficient. *p < 0.05. TMD: Temporomandibular disorders.

## 4. Discussion

Mild self-medication was the most common (89.4%) and those with painful TMD reported more often moderate self-medication.

Research has shown that pain can lead to self-medication [14]. In their preliminary study, Pastore *et al*. [8] reported that 50% of TMD patients practiced self-medication, with the most commonly used medications being analgesics, NSAIDs, and muscle relaxants. De Campos *et al*. [15] discovered that 60% of the 358 participants with TMD reported self-medication. Additionally, those with severe TMD were 4.7 times more likely to self-medicate than those with mild TMD. Once again, analgesics and anti-inflammatory drugs were the preferred medications. Afolabi *et al*. [16] found that 50% of individuals undergoing general dental treatments practiced self-medication, with analgesics being the most commonly used.

Self-medication can result from various factors, such as financial constraints, difficulty in consulting a qualified professional, and inefficacy of previously prescribed medications [14, 17]. Its potential consequences include drug resistance, adverse effects, drug interactions and even death [18]. Moreover, self-medication is typically associated with momentary relief from symptoms, which can delay the diagnosis and effective treatment of the underlying condition, leading to chronic pain [19, 20, 21]. Therefore, health promotion measures, including education on the reasonable use of self-medication and its consequences, are necessary to protect the population’s health [21].

According to Fielding *et al*. [22], there is a commonly held perception of low risk associated with the use of over-the-counter medicines such as non-opioid analgesics (dipyrone and paracetamol), and there is a need to increase public awareness of the use of these medicines in health care settings [22, 23]. The study also emphasized that pharmacists should seek relevant information from consumers to enable the provision of information regarding appropriate treatment measures [22].

A systematic review of current evidence on self-medication practices among the adult population in Brazil revealed no statistically significant differences in prevalence rates between men and women [24]. The age group included in the current study was supported by a previous cross-sectional population-based study that found that they were most likely to practice self-medication [17]. Several studies have reported higher rates of self-medication among university students compared to the general population. In Jordan, self-medication was observed in 42.5% and 78.5% of the general population and students, respectively. A meta-analysis of Iranian students showed prevalence rates of 67%, approximately 15% higher than that of the general population [25, 26]. As reported in other parts of the world, self-medication rates are higher among university students. For instance, in Uganda, the rate was 74.2% [27], in Ghana, it was 53.7% [28], in Serbia, it was 78.6% and 81.3% among medical students [29]; in Saudi Arabia, it was 63.9% [30], and in Pakistan, it was 83.0% [31].

In the present study, it was observed that females experienced painful TMD symptoms more frequently than males, which is consistent with previous research. Studies have shown that Brazilian women are more likely to experience chronic pain than men, and one study reported that women had twice the prevalence rates of general chronic pain compared to men [32, 33]. A meta-analysis of studies that included Chinese university students found a statistically significant difference in the incidence of painful and non-painful temporomandibular disorders (TMD) between genders. Females exhibited higher rates of painful TMD than males [34]. Similar results were observed in a 30-year-old New Zealand cohort, where males presented with fewer TMD symptoms than females [35]. De Campos *et al*. [15] (2019) found that 84.4% of their convenience sample of 358 participants with TMD were women. Women were also 30% more likely than men to practice self-medication.

However, the prevalence of TMD in the general population remains uncertain due to variations in study methodologies, diagnostic criteria, and demographic characteristics of the populations studied. One systematic review reported that approximately one-third of the adult population exhibited TMD, while another observational study found this proportion closer to 84% [36]. In this study, 36.9% of participants did not show any symptoms of TMD, while 48.6% reported experiencing painful TMD. The higher prevalence of TMD among university students can be attributed, in part, to the association between psychological and social factors and TMD. This population is at risk of developing psychosocial distress due to academic pressure [37, 38].

The COVID-19 pandemic may have contributed to the development of TMD symptoms due to increased anxiety. In this study, questionnaires were distributed shortly after UnB students returned to in-person classes following two years of distance learning due to pandemic restrictions. A previous study conducted with students at UnB reported higher rates of anxiety, painful TMD, and poor quality of sleep and life during the distance learning period [5]. Furthermore, the affected population continued to experience anxiety even after isolation restrictions were lifted. A study comparing fears experienced during and after isolation found increased health-related worries among young people post-lockdown [39, 40].

The study has several limitations. Firstly, it relied solely on self-reported evaluations and did not include clinical examinations, preventing a conclusive TMD diagnosis. Secondly, the study used a convenience sample, which means that students with TMD symptoms were more likely to participate in the research. Future studies investigating self-medication practices among individuals with TMD symptoms should focus on developing suitable clinical protocols and increasing awareness among professionals and patients.

## 5. Conclusions

This study’s findings indicate that while most participants had symptomatic TMD, only about 12.3% reported practicing moderate or severe self-medication. These results suggest that students with painful TMD are more likely to self-medicate than those without pain or with non-painful TMD.
